# Incorporation and Distribution of Polycyclic Aromatic Hydrocarbons in Experimental Sea-Ice

**DOI:** 10.1021/acs.est.4c13839

**Published:** 2025-04-02

**Authors:** Katarzyna Polcwiartek, Gary A. Stern, Feiyue Wang

**Affiliations:** Centre for Earth Observation Science, and Department of Environment and Geography, University of Manitoba, Winnipeg, Manitoba R3T 2N2, Canada

**Keywords:** PAHs, sea-ice, particulate organic carbon, partition coefficient, Arctic

## Abstract

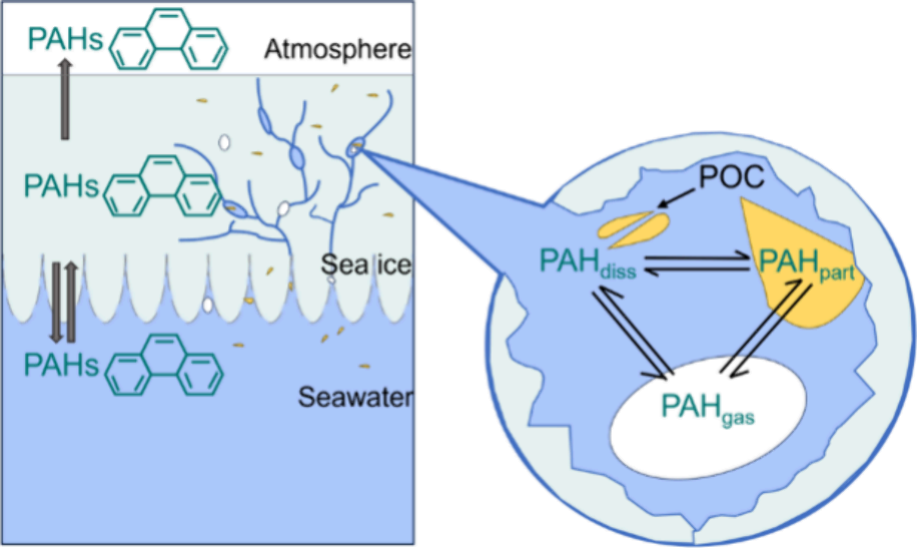

Rapid melting of sea-ice makes the Arctic more accessible for marine shipping and other industrial activities, increasing the risk of oil spills in the Arctic Ocean. Polycyclic aromatic hydrocarbons (PAHs) are among the most toxic substances in petroleum oil, yet their behavior in sea-ice-covered waters remains poorly studied. Here, we report an outdoor microcosm study to examine the partitioning behavior of four PAHs (naphthalene, phenanthrene, pyrene, and benzo(a)pyrene) across the seawater-sea-ice-atmosphere interface in the presence of particulate humic acid as a surrogate for particulate organic carbon (POC). We show that the higher the molecular weight of the PAH, the higher its concentration in sea-ice and the POC fraction. The POC-aqueous phase (seawater or bulk sea-ice) partition coefficients, *K*_d_, are reasonably well explained by temperature and salinity for all four PAHs in seawater and for phenanthrene and pyrene in sea-ice. Relationships of *K*_d_ with temperature and salinity in sea-ice and freezing seawater are complex and nonunidirectional, most likely due to the dynamic nature of sea-ice and seawater under such temperatures. This suggests that conventional equilibrium-based approaches developed for open-water conditions need to be revisited when describing the behavior of PAHs in ice-covered waters.

## Abstract 2

Scarce research exists on transport of polycyclic aromatic hydrocarbons across seawater-sea-ice-atmosphere interface. This study reports that their partitioning between particulate organic matter and the aqueous phase at or below water-freezing temperatures in the marine cryosphere deviates from that in ice-free, open-waters.

## 1 Introduction

Polycyclic aromatic hydrocarbons (PAHs) are an emerging concern in the Arctic^1,2^ due to projected increases in marine shipping^3^ and fossil fuel extraction activities as climate change makes the Arctic more accessible. Other sources of PAHs to the Arctic include tundra or forest fires in and around the Arctic and long-range transport from lower latitudes. PAHs have been shown to cause adverse effects to both Arctic sea-ice communities^4^ and marine biota.^5,6^ Yet, the role of sea-ice in the fate and transport of PAHs in the Arctic remains poorly understood.

Distribution of PAHs across the seawater-ice-air interface is governed by both physicochemical properties of PAHs and dynamic processes in the marine cryosphere, especially the movement of brine and air bubbles in sea-ice. Distribution of gaseous chemicals between sea-ice and seawater has been shown to be driven by buoyant air bubble transfer or diffusion,^7^ whereas water-soluble compounds accommodated in the brine are distributed by the brine dynamics.^8−10^ Therefore, relationships between salinity and solutes in sea-ice are used in experimental^10^ and modeling^11^ studies to assess and predict the way in which chemicals are incorporated and rejected from sea-ice. However, more hydrophobic contaminants, such as long-chain poly- and perfluoroalkyl substances, are not necessarily transported conservatively with respect to brine, likely due to their partitioning to solid ice interstitial surfaces.^10^ This may play a significant role in distribution, transformation and environmental impacts of PAHs, especially high-molecular-weight PAHs, in sea-ice containing particulate organic carbon (POC).^9,12,13^ Studies on the Antarctic sea-ice^14−16^ have shown that particle-bound metal fractions were partially immobilized on brine channel surfaces due to particle sorption to ice crystal surfaces, which separated them from brine and subsequently led to their retention in sea-ice. However, no studies have examined partitioning of organic contaminants to sea-ice POC and its impact on contaminant distribution, retention in sea-ice, and exposure pathways to the local biota.

Partitioning of PAHs between the aqueous phase and POC has implications for their toxicity, bioavailability, transport, and overall fate in the environment. It is a solubility-driven process that is quantitatively described by the partition coefficient, *K*_d_, which is affected by both physicochemical properties of the PAH and environmental parameters such as temperature and salinity.^17^ Studies have examined *K*_d_ values of PAHs in seawater at above water-freezing temperatures,^18−20^ but data are scarce for conditions relevant to polar oceans,^21^ especially in seawater and sea-ice brine at or below water-freezing temperatures. As such, it remains unknown whether the existing environmental models are sufficient to describe the transport, transformation, and effects of PAHs and other contaminants in cold environments.^9^

To fill this critical knowledge gap, here we report the results of an outdoor sea-ice microcosm experiment on the behavior of four PAHs of differing physicochemical properties [naphthalene (NAP), phenanthrene (PHE), pyrene (PYR), and benzo(a)pyrene (BaPYR)] in the presence of POC. We studied the distribution of the PAHs across the seawater-ice-air interface, examined their partitioning behavior between the dissolved and particulate phases in both the ice matrix and the underlying water column, determined their *K*_d_ values in ice and seawater, and investigated the combined effect of freezing temperature and salinity on the *K*_d_ values.

## 2 Materials and Methods

### 2.1 Experimental Setup

An outdoor sea-ice microcosm experiment was conducted from February 24 to March 16, 2020, at the University of Manitoba’s Sea-ice Environmental Research Facility (SERF) in Winnipeg, Canada. Six funnel-shaped fiberglass containers (five replicates and a control), each with a 260 L capacity (Figure S1 in the Supporting Information), were exposed to outdoor winter conditions. The experiment simulated the seasonal evolution of natural sea-ice.

Each microcosm was filled with approximately 240 L of artificial seawater formulated from groundwater and salts, with a salinity and major ion composition resembling that of standard seawater at a salinity of 33, similar to surface Arctic seawater. A detailed description of the artificial seawater preparation can be found in the study by Gao et al.^22^ The water in five of the six microcosms was spiked with 0.7 g of particulate humic acid and stirred with a rod to distribute it as evenly as possible throughout each microcosm, reaching final concentrations of approximately 2.9 mg L^–1^. Humic acid has previously been used as a surrogate for POC in studies involving snow^23,24^ and marine particles.^25^ In the current study, the particulate humic acid was prepared by mixing about 15 g of humic acid (Sigma-Aldrich) with 6 L of filtered artificial seawater from SERF, followed by filtering with 0.7 μm glass microfiber filters (GF/F, Whatman) to remove the water-soluble fraction. The particulate humic acid fraction retained on the filters was dried at 65 °C overnight and spiked for each of the five microcosms. The sixth microcosm was designated as a control and was not spiked with humic acid.

Four PAHs including NAP, PHE, PYR, and BaPYR were used in this experiment. These compounds differ greatly in molecular weight, volatility, water solubility, and other physicochemical properties (Table S1). A methanolic mixture solution containing 20 μg mL^–1^ of each of the four compounds was prepared from individual standards (Sigma-Aldrich).

Sea-ice formation in the microcosms began on February 24. Two days later (designated as day 0); after the ice in each of the microcosms had grown to a thickness of ∼8 cm, a 5 mm diameter hole was made with a hand drill through the center of the ice surface in each microcosm except the control. Subsequently, 25 mL of the PAH mixture solution was injected ∼20 cm beneath the ice–water interface using a plastic disposable pipet. This was followed by an injection of 20 mL of ambient seawater at the same depth and turning the submerged pipet tip in the underlying seawater several times to aid the dispersal of the added PAHs. The opening in the ice was resealed with ice shavings generated during the drilling. The control microcosm was not spiked with PAHs. The ice continued to grow until March 16 when the experiment was terminated. A schematic showing the experimental setup and the steps followed during the sample collection is provided in Figure S1.

During the experiment, the air temperature and wind speed were measured in situ at approximately 1.5 m above the ground using a Vaisala HMP45C probe and an UltraSonic anemometer (WindSonic), respectively.

### 2.2 Sampling Procedure

The five microcosms that were injected with POC and PAHs were treated identically but were sampled at different points in time as the ice grew. The first microcosm was sampled on February 28 (2 days post PAH injection; day 2) when the ice had grown to a thickness of 12 ± 1 cm. The other four microcosms were sampled 5, 9, 14, and 19 days post PAH injection corresponding to March 2, 6, 11, and 16, respectively. The control microcosm was sampled only on March 16.

During each sampling event, four bulk ice cores were retrieved from the corresponding microcosm using a 9 cm diameter core barrel (Kovacs Enterprise Mark II coring system). Three ice cores were dedicated to the analysis of PAHs, and the fourth core was used to determine the content of particles (i.e., POC) in the ice.

All four ice cores from each sampling event were measured for the temperature profile at a resolution of every 2 cm immediately after the ice core extraction. This was done by drilling a 5 mm diameter hole perpendicular to the core length at each depth and measuring the temperature with a digital temperature probe (Traceable). Next, all ice cores were horizontally sectioned at a vertical resolution of 3 cm using a hand saw (DeWalt) and stored in 1 L clear glass jars with aluminum foil-lined polypropylene closed-top lids in a refrigerator at 4 °C until they completely melted. The exact volumes of melted ice slices and water samples were assessed with graduated cylinders with an accuracy of 0.5 mL.

Immediately following the ice core extractions, five replicates of water samples (1 L each) were collected from the water column through the cored opening using silicone tubing and a peristaltic pump (Cole-Parmer) and stored in 1 L amber glass bottles with PTFE lined caps in a refrigerator at 4 °C for no longer than 24 h. Three of the replicate samples were dedicated to the analysis of PAHs and the other two to the measurement of particles in the water.

### 2.3 Sample Processing and Analysis

Dissolved and particulate fractions of PAHs were separated by vacuum filtration using prebaked (at 450 °C for 6 h) and preweighed 0.7 μm glass microfiber filters (Whatman). During filtration, the jars used for sample storage, as well as filtration funnels, were rinsed with ice-cold Milli-Q water (18 MΩ cm) to avoid repartitioning of PAHs between dissolved and particulate fractions. For the same reason, seawater and melted ice samples were not spiked with any organic-solvent-based standards until after filtration. To assess the filtration recoveries, independent trials of the loss of PAHs during filtration were performed in the laboratory before the experiment. The mean recoveries (*n* = 6) for the four PAHs were 73 ± 15%, 87 ± 8%, 84 ± 11%, and 91 ± 13%, respectively (Table S2).

Following the filtration, liquid and particulate fractions were spiked with the deuterated PAH surrogate standards (NAP-D_8_, PHE-D_10_, PYR-D_10_, and BaPYR-D_12_) in methanolic solution (Sigma-Aldrich) and in iso-octane solution (Cambridge Isotope Laboratories), respectively. Solid-phase extraction (SPE) and ultrasonification were then performed to extract the PAHs from dissolved and particulate fractions, respectively, following established procedures^26,27^ with slight modifications, as detailed in Text S1. Following the SPE procedure, 20 mL aliquots of effluent were collected into 50 mL vortex tubes for bulk ice salinity measurements using an Orion Star A212 conductivity meter (Thermo Scientific).

Ice cores and water samples for the particle concentration measurements were filtered through prebaked and preweighed 0.7 μm glass microfiber filters. The particulate humic acid mass was determined by gravimetry after drying the GF/F in an air-heated oven at 55 °C until constant weight and equilibration at room temperature in a desiccator.

The analysis of PAHs was performed on a gas chromatograph (Agilent 7890B) coupled with a triple quadrupole mass spectrometer (Agilent 7010B) equipped with a PAL RSI 85 autosampler, a Rxi-PAH analytical column (60 m × 250 μm × 0.1 μm), and helium as the carrier gas. Information regarding the details of the instrumental analysis is presented in Text S2. Quantification of PAHs was achieved with a set of external standards (Restek) containing native and deuterated NAP, PHE, PYR, and BaPYR with concentrations ranging from 0.488 to 1000 ng mL^–1^. A detailed description of the targeted ions is provided in Table S3. The QA/QC information concerning the blanks and instrumental analysis is provided in Text S3.

### 2.4 Data Analysis

The brine volume fraction (*V*_B_) of the respective sea-ice samples was calculated from the sea-ice temperature (*T*) and bulk ice salinity (*S*) based on the formula of Cox and Weeks.^28^ The ice growth rate was calculated by dividing the ice thickness growth (centimeters) between the two consecutive measurement dates and the period the growth occurred (day).

The partition coefficients (*K*_d_; L kg^–1^) for NAP, PHE, PYR, and BaPYR in both ice and water matrices were calculated as the ratio of their concentrations in the particulate humic acid phase (ng kg^–1^) to those in the dissolved aqueous phase (liquid phase obtained after filtration) of seawater or melted bulk ice samples (ng L^–1^).

The temperature and salinity dependence of *K*_d_ was examined by fitting the experimental data to the following general equation 1(29):
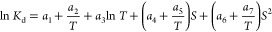
1where *T* is temperature (K), *S* is salinity, and *a_i_* are fitting parameters.

Statistical analysis was carried out using SigmaPlot 14.0 (Systat Software Inc.). Student’s *t* test and one-way ANOVA were used to examine the statistical difference under various conditions, and the significant level was set at 0.05 for each test. Regression analyses were used to determine the relationships between PAH concentrations and bulk ice salinity.

## 3 Results

### 3.1 Sea-Ice and Seawater Properties

Throughout the study, the ambient air temperature fluctuated between −19 and +6 °C, mostly well below 0 °C; the daytime high reached above 0 °C only on 4 days (February 24, and March 1, 7, and 11) (Figure S2a). The wind speed varied between 0.2 and 5.1 m s^–1^ with an average of 1.1 m s^–1^ (Figure S2b).

Despite the ambient air temperature variability, the ice continued to grow throughout the experiment. The ice growth rate was 4.0 cm day^–1^ between February 24 and 26 and decreased to between 0.1 and 2.3 cm day^–1^ on subsequent days (Table S9). The ice thickness ranged from approximately 8.0 ± 0.4 to 19.3 ± 3.8 cm on day 0 and day 19, respectively. There were no major snowfall events during the experiment.

The ice was relatively warm throughout, with the average temperatures at the surface and bottom of the ice reaching −2.7 ± 1.5 and −2.5 ± 0.9 °C, respectively. The temperature of sea-ice on day 2 (Figure S3) increased steadily from the ice surface to the ice–water interface. The ice temperature for the subsequent dates showed an isothermal profile throughout the ice column as the ambient air temperature rose (Figure S2).

While the ice temperature increased over time, the average bulk ice salinity decreased from 10.1 ± 1.2 on day 2 to 6.3 ± 0.3 on day 19. The underlying seawater salinity varied between 36.2 ± 3.0 and 45.1 ± 1.4 during the study (Figure S3b). The bulk ice salinity exhibited a C-shaped profile with slightly higher salinities at the top- and bottom-most parts of the ice in relation to the middle ice sections (Figure S3b), as commonly observed in young and growing natural sea-ice.^30^

The maximum particulate humic acid concentration reached 7.1 ± 1.6 mg L^–1^ at the ice surface; it gradually decreased with ice depth to 1.9 ± 0.9 mg L^–1^ in the bottom ice layers (Figure S3c). The mean particulate humic acid concentration in seawater was 2.9 ± 0.1 mg L^–1^. Mass budget calculations show a total loss of up to 8–11% of the particulate humic acid in the microcosms, likely due to a combination of factors such as sorption onto the container walls and settling to the bottom.

The brine volume fraction, *V*_B_, in sea-ice increased from the surface (11–31%) toward the ice–water boundary layer (19–37%), as the ice warmed up with depth (Figure S3d). As these values well exceeded the critical ice porosity threshold of 5%^31,32^ in the entire thickness, movement of fluids was expected within the ice matrix throughout the experiment.

### 3.2 Distribution of PAHs between Seawater, Sea-Ice and Atmosphere

**Figure 1:**
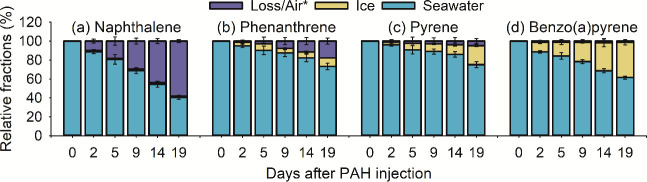
Distribution of the four PAHs between the water, sea-ice, and air compartments. The loss to the air was estimated from the difference between the initial mass on day 0 and the sum of the seawater and sea-ice fractions on each sampling day.

The total amounts of each PAH in the microcosms were calculated as the sum of their quantities measured in the dissolved and particulate fractions of bulk sea-ice and the water column (Table S6). Percentages of the PAHs in each of the microcosm compartments and potential losses (Figure 1) were calculated relative to the original amounts added (day 0), which ranged from 498 μg for PYR to 511 μg for NAP (Table S6). As can be seen from Figure 1 and Table S6, quantities of NAP, PHE, and PYR in the microcosms gradually and significantly (*p* < 0.05) decreased with time; by the end of the study, the losses observed for NAP, PHE, and PYR had reached 58, 17, and 5% of their initial masses, respectively. In contrast, there was no significant loss (*p* > 0.05) of BaPYR. The lost portions of each compound were assumed to be fractions volatilized in the air and were calculated as a difference between the compound’s initial mass on day 0 and the sum of the seawater and sea-ice fractions. The concentrations of PAHs in the air just above the microcosms were not measured in this study.

**Figure 2:**
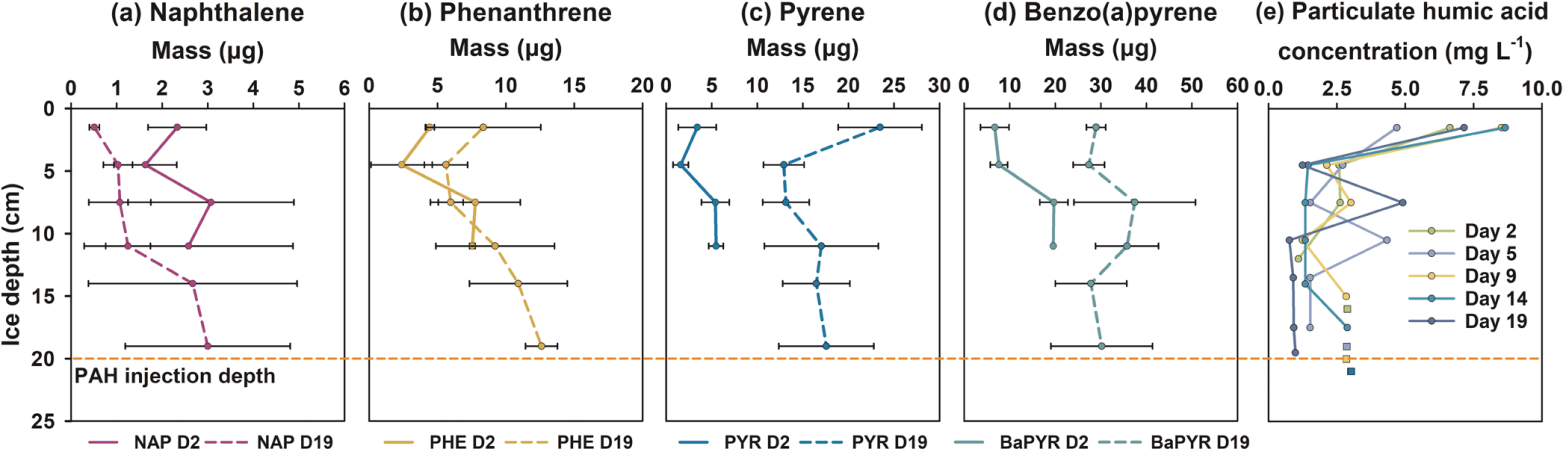
Vertical distribution profiles of (a–d) PAHs in sea-ice on day 2 (solid line) and day 19 (dashed line) and (e) concentrations of particulate humic acid throughout the experiment. The square symbols in (e) indicate particulate humic acid concentrations in the water column.

Examples of vertical distribution profiles of the four PAHs in the ice are shown in Figure 2; vertical profiles for all of the sampling events can be found in Figure S4. Overall, the higher the molecular weight of the compound, the larger its quantity is encased in the ice. For instance, on day 2, up to 9.6, 22.1, 15.9, and 53.7 μg of NAP, PHE, PYR, and BaPYR, respectively, were found in the ice, with the largest quantity of each compound found at the ice depth corresponding to the ice–water interface at the time of PAH injection (∼8 cm) and below. Over time, the quantities of PHE, PYR, and BaPYR in the ice significantly (*p* < 0.05) increased. No change in the content of NAP (*p* > 0.05) was observed (Figures 1 and 2). On day 19, predominant portions of NAP and PHE were present in the bottom-most ice sections (below 11 cm), whereas PYR and BaPYR were more dominant in the middle and top sections (above 11 cm).

### 3.3 Partitioning of PAHs between Liquid and Particulate Phases

**Figure 3:**
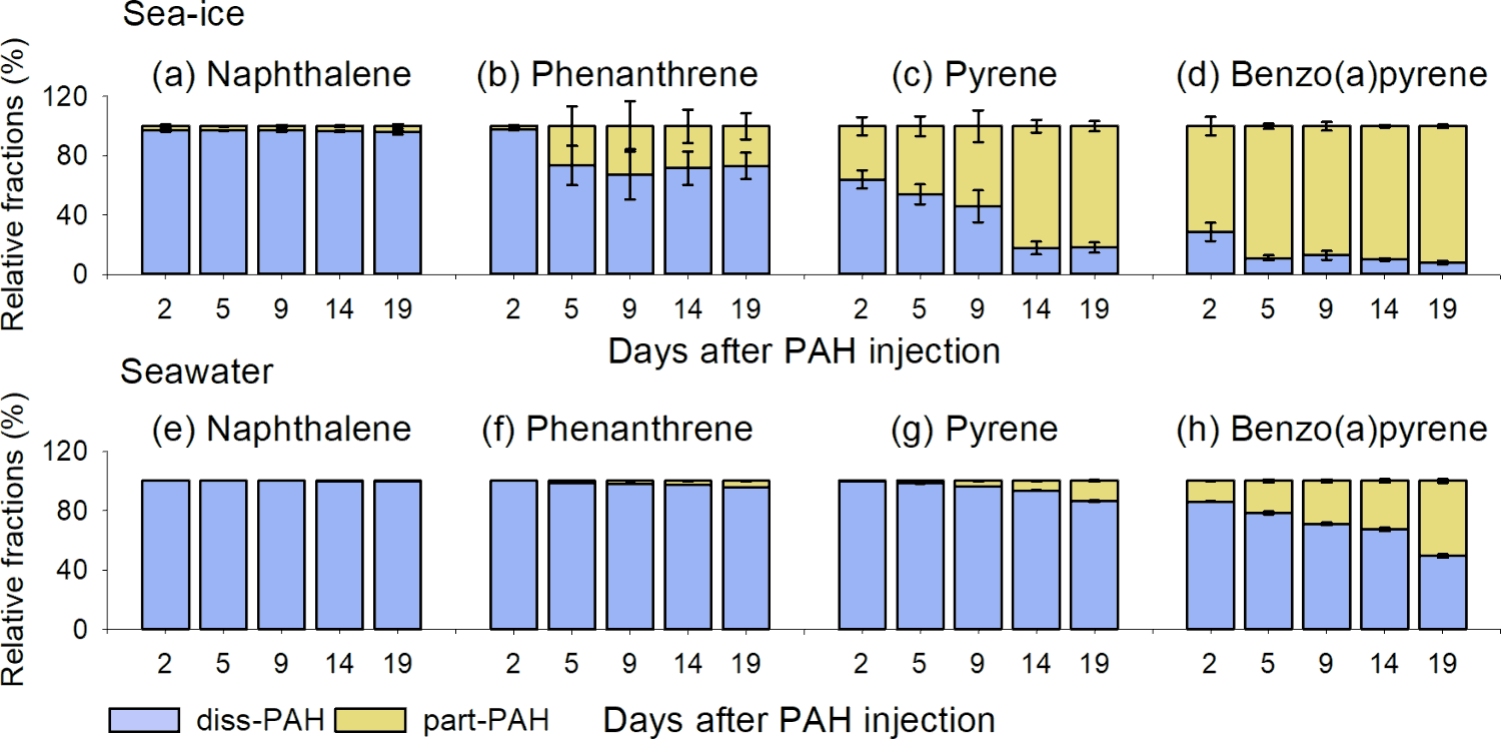
Relative % of PAHs detected in dissolved (diss-PAH) and particulate (part-PAH) phases in the ice (a–d) and the underlying water column (e–h). The standard deviations for the four PAHs in seawater are close to 0, and hence, the error bars are not visible.

Measurements of the PAH masses in the dissolved (diss-PAH) and particulate (part-PAH; i.e., PAHs bound to particulate humic acid) fractions allow for a better evaluation of their fate and exposure in the studied system (Figure 3). In the ice, up to 3, 4, 36, and 71% of NAP, PHE, PYR and BaPYR, respectively, were bound to humic acid on day 2. By day 19, the corresponding part-PAH fractions significantly (*p* < 0.05) increased to 4, 27, 82, and 92%.

In the underlying seawater, the four PAHs bound to particulate humic acid followed a similar pattern (Figure 3b), where the higher the compound’s molecular weight, the larger its content in the part-PAH fraction. For example, the part-PAH fractions of BaPYR in the water were 0, 0.1, 0.4, and 14% on day 2 and significantly (*p* < 0.05) increased to 0.1, 4, 14, and 51% on day 19.

The mean log *K*_d_ values for the studied PAHs calculated from the PAH concentrations in the dissolved and particulate fractions in sea-ice on day 2 were 3.8, 3.7, 5.1, and 5.8 for NAP, PHE, PYR, and BaPYR, respectively, and by day 19, they significantly (*p* < 0.05) increased to 4.5, 5.7, 6.3, and 6.7, respectively (Table S7).

## 4 Discussion

### 4.1 Microcosm Approach for Studying PAH Partitioning in the Sea-Ice Environment

Partitioning of PAHs in a sea-ice environment can be studied at field and laboratory scales. While field studies reflect real-world situations, they are logistically challenging, and it is difficult to isolate a specific process that is being studied due to the dynamic nature of the real-world environment. On the contrary, the laboratory-based approach allows greater control over experimental conditions, which leads to more precise data, but may not be representative of real-world situations, which can limit the applicability of the findings.

In this study, we used a new microcosm approach that bridges between the field and laboratory approaches, allowing us to isolate and probe partitioning of PAHs among different phases (dissolved vs particulate) in different compartments (sea-ice and seawater) under ambient winter conditions that resemble the Arctic. This approach is particularly useful due to a much simpler operational and less costly setup than the field-scale approach, the ability to control the composition of seawater (e.g., salinity, the type and concentration of POC, and the choice and concentration of the studied PAHs), and the ability to control the timing of PAH injection. One of the limitations of this approach, however, is a lack of mixing in the microcosms, which prevented efficient circulation of seawater and, consequently, homogeneous distribution of POC and PAHs.

As shown in Figure S3, the properties of the ice from the microcosms resemble those of natural sea-ice. Specifically, the observed temperature gradient from the surface to the bottom of the ice is characteristic of natural sea-ice. The bulk ice salinity profiles of the ice grown during our study are representative of young and growing sea-ice with the characteristic C-shape curve with higher salinities at both interfaces. This is due to the rejection of highly saline brine during the ice formation and growth processes that subsequently accumulate at the surface of the ice and the underlying water column.^30,33^ Due to the small volume of the microcosm, this resulted in a much higher salinity in the underlying seawater.

Mass balance calculations were conducted to assess the potential loss of PAHs in the microcosms and the potential experimental artifacts. The observation that the total amount of BaPYR, the least volatile among the studied PAHs, remained essentially unchanged (*p* > 0.05) throughout the study period (Figure 1 and Table S6) confirms negligible losses of the compound due to sorption to the sides of the microcosms. Moreover, control samples and field blanks contained very low levels of PAH (from not detectable to 1.5 ng L^–1^; Table S4), confirming that atmospheric contamination of PAHs can be ruled out and the chemicals detected in the samples originated from the PAH spike solution. Biodegradation of PAHs by microbial organisms is known to occur in the natural snow and ice environment.^34^ Since SERF employs artificial seawater formulated at the beginning of the winter, the microbial activity in SERF seawater and sea-ice has been shown to be very low during winter months^22,35^ and with negligible biodegradation potential for PAHs.^36^

### 4.2 Incorporation and Early Transport of PAHs across the Seawater-Sea-Ice-Atmosphere Interface

Chemicals introduced into the multiple-phased sea-ice environment will partition to different phases (gaseous, dissolved, and particulate) based on their physicochemical properties. Once accommodated in a preferential compartment (ice crystals, brine or air bubbles, and organic matter), they are subsequently transported from within and out of the ice (i.e., atmosphere and/or seawater) via the ice internal processes^9,30,33^ (see Text S4). For chemicals in ice-covered seawater to reach the ice surface, a pathway through a succession of connected pores (air and brine inclusions) must connect the seawater to the air-ice interface. As a result, the transport of chemicals occurs through buoyant air bubble transfer and/or concentration gradient-driven diffusion in brine channels,^7,37^ brine movements,^8^ as well as sorption to the solid brine channel and ice interstitial surfaces.^10,11^

Following the injection (day 0), all PAHs were present exclusively in the water column (Figure 1). Their presence in the ice on day 2 indicates their rapid incorporation within the growing sea-ice through freeze rejection (dissolved fraction) and particle entrapment (via partitioning onto particulate humic acid; particulate fraction), as well as the formation of bubbles (gaseous fraction).^9^

Since *V*_B_ exceeded 5% (Figure S3) throughout the ice, the bulk ice phase was expected to be permeable,^31,32^ and thus the water–air exchange of the PAHs was possible. This is confirmed by the vertical profiles (Figure 2), showing that all four compounds were detected across the entire ice thickness. The differences in the quantities and vertical distribution profiles suggest that the four PAHs were subjected to different transport processes, which stem from their varying physicochemical properties.

Specifically, because of its relatively high vapor pressure and water-solubility (Table S1), NAP would have preferentially partitioned to air inclusions in the ice or dissolved in the liquid brine. Subsequently, it migrated to the air-ice interface via buoyant air bubble transfer and/or concentration gradient-driven diffusion in brine channels, similar to the transport of other gases in sea-ice.^7,37^ NAP could also be expelled from the growing ice to the air-ice interface due to freeze rejection.^38^ Vertical transport of NAP to the air-ice interface and its subsequent release to the atmosphere could explain the considerable loss of NAP^39,40^ that occurred early in the experiment (Figure 1) due to its high vapor pressure.

The lower evaporative losses observed for the remaining PAHs (Figure 1) can be attributed to their relatively lower vapor pressures (Table S1). Therefore, bubble dynamics would be less important for their movement through the ice. Instead, their vertical transport would be partially controlled by brine migration depending on their solubility in the brine^8,40−42^ and partially by partitioning to the particulate humic acid depending on their affinity to particulate matter.

Brine convection, usually responsible for the transport of solutes in brine channels, appeared to regulate the distribution of the four PAHs to a limited extent only. Specifically, the mass distribution profiles of the four PAHs exhibit different patterns than the C-shaped profile expected for salinity and major sea salts (Figure 2); the concentrations of the four PAHs exhibited poor relationships (*R*^2^ = 0.13–0.42) with bulk ice salinity (Figure S5). These results suggest that the behavior of PAHs in the experimental ice deviates from that of major sea salts.

Given that particulate matter in sea-ice is much less mobile,^43,44^ the particle-associated PAHs are not expected to be readily displaced out of the ice. Sorption onto the particulate humic acid is attributed to hydrophobic properties of a chemical, often expressed as the octanol–water partition coefficient (*K*_ow_)^17^ (Table S1) that increases with increasing molecular weight of the PAHs. This is supported by the molecular-size-dependent distribution patterns in their *K*_d_ values (Table S7). We observe that the higher the molecular weight of the compound, the larger its portion in the ice (Figure 1) and the particulate humic acid fraction (Figure 3). This causes higher retention of high-molecular-weight PAHs in the ice relative to low-molecular-weight PAHs (Figure 1) and potentially their delayed release from melting ice, just like reported previously for snow.^23^

### 4.3 Effect of Temperature and Salinity on Partitioning of PAHs between the Liquid and Particulate Phases

Partitioning of PAHs between POC and water is dependent on both temperature and salinity. At above the water-freezing temperature, the *K*_d_ values of PAHs are commonly affected by the combination of these two in a way that *K*_d_ increases with decreasing temperature and increasing salinity.^17^ Reitsma et al.^45^ appears to be the only study that has examined temperature and salinity dependence of PAH partitioning under water-freezing conditions. They reported that low-molecular-weight PAHs attained thermodynamic equilibrium in a passive sampler-water system and showed that the respective *K*_d_ values increased as the temperature decreased from +20 to −15 °C and salinity increased from 0 to 245, which agrees with the patterns at above water-freezing temperatures. In contrast, *K*_d_ values of the high-molecular-weight PAHs tended to decrease at temperatures colder than −4 °C and salinity higher than 100, which resulted from the high-molecular-weight PAHs being unable to equilibrate in saline brines below 0 °C. Extremely slow equilibration of the high-molecular-weight PAHs results from increased water viscosity at colder and more saline conditions.^45^

**Figure 4:**
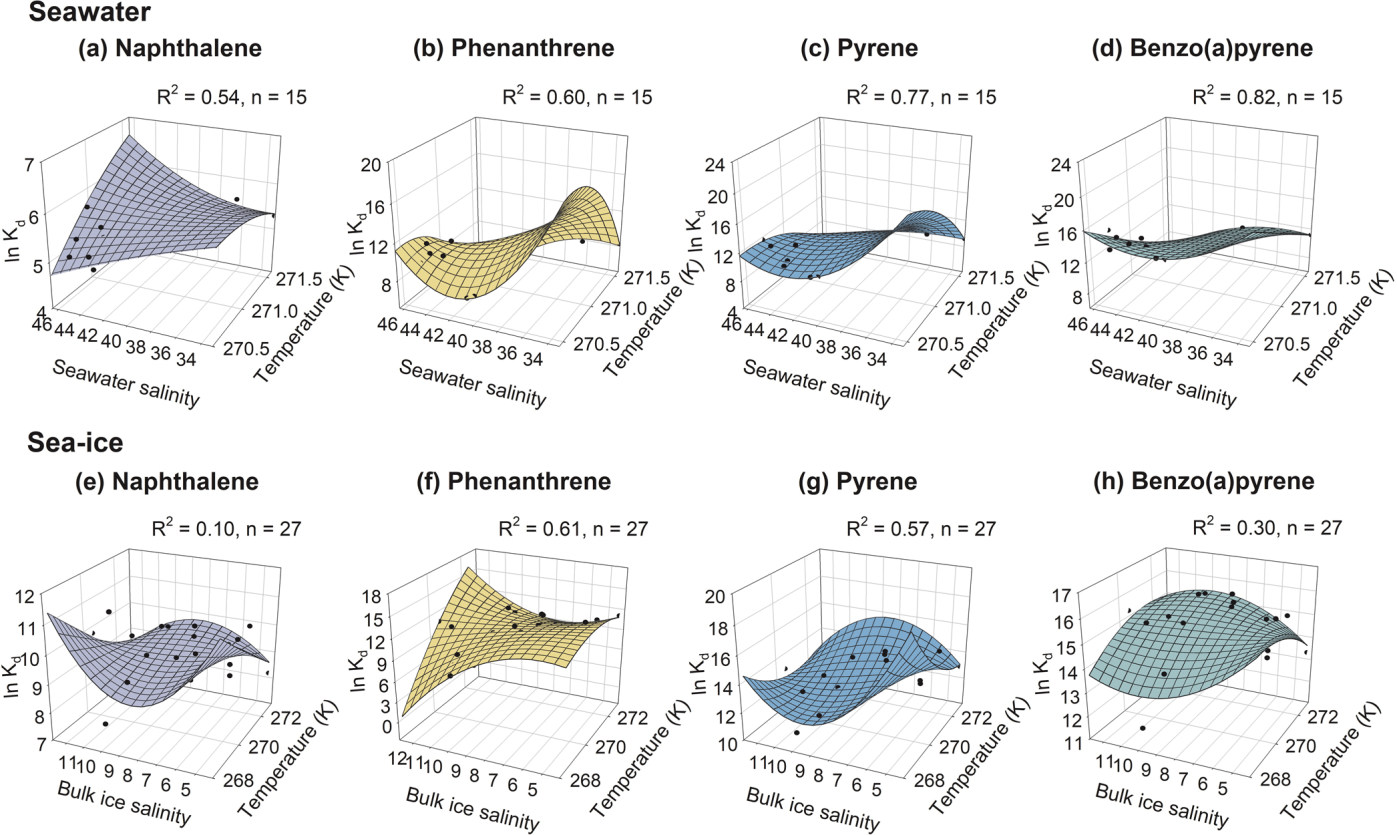
Best-fit plots of the dependence of PAH partition coefficients on temperature and salinity in seawater (top panels) and sea-ice (bottom panels).

Our study provides a unique opportunity to examine whether such a temperature and salinity dependence of *K*_d_ applies to seawater or sea-ice at or below water-freezing temperatures; the results are shown in Figure 4 and Table 1. For PAHs partitioning between particulate humic acid and seawater, the four PAHs showed a moderate-to-strong relationship with temperature and salinity (*R*^2^ ≥ 0.54) over a very narrow freezing-temperature range of 270.4 to 271.6 K and the salinity range of 33.1 to 46.4 (Figure 4a–d). In bulk sea-ice, *K*_d_ values for the two intermediate-molecular-weight PAHs, PHE and PYR that are moderately water-soluble, are also reasonably well explained by the temperature and salinity dependence (*R*^2^ ≥ 0.57) over a temperature range of 268.2 to 272.5 K and bulk ice salinity range of 4.2 to 12.8 (Figure 4f,g). However, the fitting for the lowest-molecular-weight PAH, NAP, was the poorest (*R*^2^ = 0.10; Figure 4e) due to its very high volatility and thus large evaporative loss from the bulk sea-ice (Figure 1a). The fitting for the heaviest-molecular-weight PAH, BaPYR, was also poor (*R*^2^ = 0.30; Figure 4h), likely due to its very low water solubility and high hydrophobicity and propensity to solid ice surfaces.

**Table 1:** Dependence on Temperature (*T*) and Salinity (*S*) of the Partition Coefficient (*K*_d_) of PAHs between Particulate Humic Acid and Seawater or Sea-Ice, as Fitted with the Following Equation:

matrix	PAH	*a*_1_	*a*_2_	*a*_3_	*a*_4_	*a*_5_	*a*_6_	*a*_7_
seawater (*T*: 270.4–271.6 K, *S*: 33.1–46.4)	NAP	5.967 × 10^5^	–2.493 × 10^7^	–9.008 × 10^4^	–1.403 × 10^2^	3.797 × 10^4^	2.358	–6.383 × 10^2^
	PHE	1.035 × 10^7^	–4.054 × 10^8^	–1.580 × 10^6^	4.228 × 10^3^	–1.147 × 10^6^	–5.331 × 10^1^	1.446 × 10^4^
	PYR	5.832 × 10^6^	–2.254 × 10^8^	–8.925 × 10^5^	2.919 × 10^3^	–7.921 × 10^5^	–3.552 × 10^1^	9.638 × 10^3^
	BaPYR	–3.618 × 10^3^	1.042 × 10^7^	–6.210 × 10^3^	2.235 × 10^3^	–6.060 × 10^5^	–2.859 × 10^1^	7.752 × 10^3^
sea-ice (*T*: 268.2–272.5, *S*: 4.2–12.8)	PHE	3.006 × 10^4^	–1.251 × 10^7^	–4.541 × 10^4^	–3.260 × 10^2^	8.803 × 10^4^	2.602 × 10^1^	–7.037 × 10^3^
	PYR	–2.422 × 10^5^	1.035 × 10^7^	3.642 × 10^4^	4.704 × 10^2^	–1.274 × 10^5^	–2.614 × 10^1^	7.077 × 10^3^

Unlike in sea-ice, the distribution of the four PAHs in seawater is not impacted as much by their partitioning to a gaseous phase (e.g., air inclusions) or sorption to solid surfaces different than particulate humic acid (e.g., ice crystal surfaces) that occur simultaneously with the POC-water partitioning process. This is due to the lower complexity and greater homogeneity of the seawater environment. We believe this is the reason why the *K*_d_ values in seawater exhibit stronger relationships with temperature and salinity than those in sea-ice.

Overall, the dependence of *K*_d_ values on temperature and salinity in seawater and sea-ice is more complex than that at above water-freezing temperatures. Different from the well-observed unidirectional influence of temperature or salinity on *K*_d_ values at above-freezing temperatures,^17^ decreasing sea-ice or seawater temperature and increasing salinity could result in enhancing or diminishing *K*_d_ values, depending on specific temperature-salinity conditions. This is evident in the surface plots that have more than one slope and multiple distinctive peaks (Figure 4).

One reason for the deviation from the expected temperature and salinity dependence of *K*_d_ in sea-ice could be due to the fact that a thermodynamic equilibrium between PAHs in particulate humic acid and in bulk sea-ice could not be truly established in an extremely dynamic sea-ice environment where the volume and boundaries of ice, brine, and air bubbles are always changing due to diurnal and seasonal changes of ambient air temperature (Figure S2), as suggested by Garnett et al.^10^ Similarly, the dynamically changing salinity of the underlying seawater due to the ongoing brine drainage could hinder the equilibration of PAHs in seawater, despite small temperature changes. Moreover, thermodynamic equilibrium could have been furt delayed by enhanced viscosity of the ice fluids and seawater under colder and saltier conditions, which, in turn, impeded diffusion of the PAHs, similar to that reported for the passive sampler-water systems.^45^ Another study shows that even after 12 weeks, high-molecular-weight PAHs were not able to reach equilibrium in a passive sampler-water system at near-freezing temperature and salinity of seawater.^46^

These results stress the challenges of determining the POC-water equilibrium *K*_d_ of PAHs under dynamically changing cold temperatures, high salinity, and ambient conditions. Hence, thermodynamic equilibrium-based parameters, such as *K*_d_, may be of limited use when describing or modeling PAH behavior in the dynamic sea-ice environment or seawater at below-freezing temperatures, where common theoretical principles established for temperatures above 0 °C may not apply.

### 4.4 Evolution of the PAH Behavior across the Seawater-Sea-Ice-Atmosphere Interface

**Figure 5:**
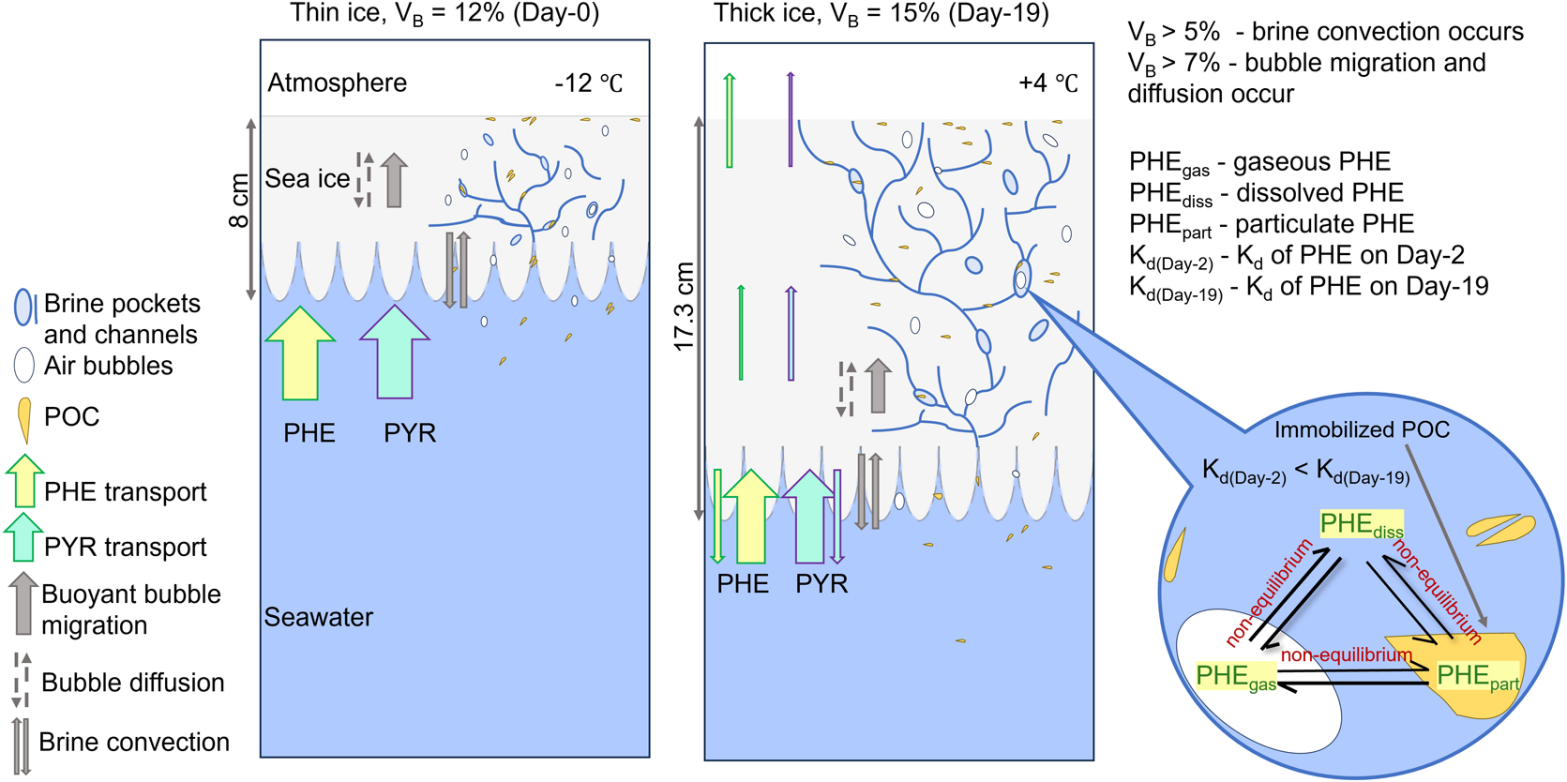
Schematic summary of the physical and chemical processes driving the distribution of PAHs in the microcosms using phenanthrene (PHE) and pyrene (PYR) as examples. *V*_B_: brine volume fraction. *K*_d_: partition coefficient between the particulate and dissolved phases. POC: particulate organic carbon.

Based on the above results, a general evolution scheme of PAHs across the seawater-sea-ice-atmosphere interface is depicted in Figure 5. As the ambient air temperature increases with time (Figure S2), the ice warms up and becomes more porous (Figure S3) due to expanding brine and air inclusions.^32^ As a result, the evaporative loss of the low-molecular-weight PAHs increases (*p* < 0.05) with time, while brine-associated PAHs are more efficiently flushed to the underlying seawater with circulating brine.

PAHs present in the ice are not limited to brine channels but rather distributed throughout the bulk ice through chemical-specific processes, such as partitioning to the dissolved phase (e.g., ice brine),^8^ partitioning to solid phases (e.g., ice interstitial surfaces or particulate humic acid),^10,14−16^ or partitioning toward the gaseous phase (e.g., air bubbles). As the ice grows, its volume, phase content, and properties (e.g., temperature and salinity) change rapidly and dynamically. Therefore, PAHs that partition between these phases are unlikely to reach true thermodynamic equilibrium. With time, the ice becomes warmer and less saline, which promotes an overall increase (*p* < 0.05) in partitioning of the intermediate- and high-molecular-weight PAHs to the particulate humic acid (Figure 3, Table S7). While the partitioning behavior of PAHs of intermediate water solubility, such as PHE and PYR, appear to be relatively well explained by evolving temperature and salinity of the ice, PAHs of more extreme properties, such as NAP and BaPYR, behave in a way that is poorly explained by temperature and salinity.

The enhanced partitioning of PAHs to the particulate humic acid leads to an increasing fraction of intermediate- and high-molecular-weight PAHs in the ice and their subsequent retention. The retention of the high-molecular-weight PAHs in the ice may potentially increase exposure to any ice-dwelling biota.^8,10^ This is particularly important when sediment-laden ice becomes more common in the Arctic^12^ and seasonal ice becomes enriched with organic matter from algal blooms, both having the potential to scavenge and interact with organic contaminants.^9,47^ Furthermore, while particulate humic acid was used as a surrogate for POC in this study, it should be noted that the presence of other particulate matter, for instance, soot, may further enhance the retention of PAHs in sea-ice. This is because of much higher affinities of PAHs to soot.^48,49^ As shipping activities and tundra and forest fires could readily deposit soot onto snow and sea-ice, the interactions of soot with PAHs as well as with other contaminants in the sea-ice environment need to be considered in future studies.

## Supporting Information Available

Additional information on quality assurance and quality control; SPE and ultrasonication procedures; instrumental method; brine dynamics in sea-ice; physicochemical properties of the four PAHs; schematic of the experimental design; and other relevant results from the study (PDF)
