# Supplementary material for: Incorporation and Distribution of Polycyclic Aromatic Hydrocarbons in Experimental Sea-Ice
Source: Environ Sci Technol. 2025 Apr 2;59(14):7310–9. doi: 10.1021/acs.est.4c13839 (PMC12005189; doi:10.1021/acs.est.4c13839)
Supplement: Supplementary file 1 — es4c13839_si_001.pdf [file es4c13839_si_001.pdf]

Supporting Information

Incorporation and Distribution of Polycyclic Aromatic Hydrocarbons in Experimental Sea-Ice

Katarzyna Polcwiartek, Gary A. Stern, and Feiyue Wang*

Centre for Earth Observation Science, and Department of Environment and Geography, University of Manitoba, Winnipeg, MB, R3T 2N2, Canada

Correspondence to: Feiyue Wang (feiyue.wang@umanitoba.ca)

Number of pages: 12

Number of additional text sections: 4

Number of tables: 8

Number of figures: 5

Table of Contents

Text S1. Solid-phase extraction and ultra-sonication procedures

Text S2. Instrumental analytical method details

Text S3. Quality assurance and quality control

Text S4. Brine dynamics in sea ice

Table S1. Physical-chemical properties of the selected PAHs at freezing temperatures.

Table S2. Filtration recoveries.

Table S3. Ions targeted during the GC-MS analysis with the SIM mode.

Table S4. Background concentrations of PAHs and particles in the microcosms and blanks.

Table S5. Solid-phase extraction and ultra-sonication recoveries and method detection limits.

Table S6. Mean and standard deviations of total masses of the four PAHs throughout the experiment.

Table S7. Mean apparent distribution coefficients and standard deviations for the four PAHs in the ice and the seawater media over time.

Table S8. Average ice thickness and ice growth rate throughout the experiment.

Figure S1. Schematic of the experimental design, key steps of the study, and sample collection plan, and a picture of the microcosm set-up.

Figure S2. Variabilities of the in situ ambient air temperature and wind speed during the experiment, as measured at 1.5 m above the ground.

Figure S3. Vertical profiles of temperature, bulk salinity, concentration of particulate humic acid (HA), and brine volume fraction across the bulk ice and in the water column in the experimental microcosms at various days after the addition of particulate HA and PAHs.

Figure S4. Vertical profiles of the respective PAHs in the ice column throughout the experiment.

Figure S5. Relationship between the bulk salinity of sea ice and the total concentrations of the PAHs in young experimental sea ice on Day 2.

Text S1. Solid-phase extraction and ultra-sonication procedures

Liquid fractions obtained upon filtration were loaded onto solid-phase extraction (SPE) cartridges (Sep-Pak C18, 3cc, 500 mg sorbent, Waters) pre-conditioned with methanol (Thermo Scientific) and equilibrated with a 40% aqueous solution of methanol. Following the sample loading step, the cartridges were rinsed with MQ water and dried with N_2. The 4 PAH compounds were eluted off the cartridge using 5 mL of hexane (Thermo Scientific). The extracts were evaporated to 0.5 mL using a rotary evaporator, then solvent-exchanged to 1 mL of iso-octane (Thermo Scientific).

Following the filtration, the glass microfiber filters were placed into 20 mL amber glass vials and stored in a fridge at 4 °C. Sample extraction was done using 15 mL of dichloromethane (Thermo Scientific) and subsequent ultra-sonification of the vials was conducted in an ultrasonicating bath (Branson 1800) for 30 min. Solvents from the vials were filtered through 0.45 μm PTFE membrane syringe filters to separate extracts with accommodated PAHs and the GF/F filter residue. Next, the extracts were concentrated to 0.5 mL and solvent-exchanged to 1 mL of iso-octane. The extracts resulting from both SPE and ultra-sonification were stored in a freezer at –20 °C until the analysis.

Text S2. Instrumental analytical method details

The analysis of PAHs was performed on a gas chromatograph (Agilent 7890B) coupled with a triple quadrupole mass spectrometer (Agilent 7010B) equipped with a PAL RSI 85 autosampler, a Rxi-PAH analytical column (60 m×250 μm×0.1 μm) and helium as the carrier gas. The GC-MS was operated in the single ion monitoring (SIM) mode. A sample volume of 1 μL was injected in splitless mode. The electron ionization energy was 70 eV. The oven regime was set as

follows: 40 °C (1 min hold), 30 °C min^{-1} to 140 °C, 5 °C min^{-1} to 180 °C, and then 10 °C min^{-1} to 320 °C with a 10 min hold.

Text S3. Quality assurance and quality control (QA/QC)

Seawater (1 L, n = 2) was collected from each experimental microcosm on February 24, before the injection of HA and PAHs, to determine background concentrations of particles and PAHs in the artificial seawater. The four PAHs were not detected in the seawater, and the average concentration of background particles was 0.7 ± 0.2 mg L^{-1}. Seawater and bulk ice samples were also retrieved from the control microcosm to monitor background concentrations of PAHs (n = 3 each for water and ice) and particles (n = 3 each for water and ice). Background pyrene concentration in the bulk ice blanks was 0.8 ± 0.2 ng L^{-1}, whereas the remaining PAHs were not detected in either control seawater or sea-ice. The background particle concentrations in the control seawater and ice were 0.6 ± 0.3 mg L^{-1} and 0.9 ± 0.4 mg L^{-1}, respectively. Field blanks (1 L of Milli-Q water (MQ, n = 4) and 1 L of artificial seawater from the main SERF pool, n = 3) and laboratory blanks (1 L of MQ water, n = 14) were collected throughout the sampling events and sample processing, respectively, to assess potential contamination. The concentrations of PAHs in the field and laboratory blanks ranged from 1.1 to 1.5 ng L^{-1} and from 0.6 to 1.3 ng L^{-1}, respectively (see Table S4). The particles in the corresponding blanks were not detected. Blank samples were processed following the same protocols as the regular samples.

The recoveries of the solid phase extraction (SPE) and ultra-sonification extraction of PAHs were monitored by the surrogated mixture of deuterated PAHs added prior to the extraction. The recoveries of deuterated standards reached 24, 67, 75 and 91% for NAP, PHE, PYR and BaPYR, respectively for the liquid fractions, whereas the recoveries for the particulate fractions

reached 77, 87, 95 and 101% for the corresponding compounds, respectively (Table S5). Since the recovery values for the respective deuterated PAHs were consistent within the population of samples, the recoveries are assessed acceptable. Low recoveries observed for naphthalene could be attributed to its high volatility.

Reported concentrations of all compounds were recovery corrected. Pre-concentrated extracts were also spiked with 2-fluorobiphenyl (Restek) as a recovery standard to compensate for variances in the final extract volume and injection volume.

The external calibration curves for each compound contained at least 10 concentrations and exhibited R^2 values higher than 0.99. The method detection limits (MDLs), calculated based on U.S.EPA (2016), ranged from 0.9 to 2.1 ng L^{-1} (Table S5).

Text S4. Brine dynamics in sea ice

Movement of liquids (brine) in sea ice can occur through brine rejection, convective drainage, flushing, as well as upward migration to the snow.[1-3] During ice formation and growth, salts are rejected from the ice matrix to the underlying seawater due to size restrictions throughout a process of brine rejection (freeze rejection). On the other hand, convective drainage occurs when more concentrated and denser brine in the ice is replaced with the adjacent less saline seawater at the base of the ice. Brine flushing is a gravity-mediated process and is a result of surface ice melt or the presence of snow. In addition, brine can also migrate upwards through the ice to the overlying snow.

Table S1. Physicochemical properties of the four studied PAHs at freezing temperatures.

PAH	Molecular weight ($g\ mol^{-1}$)	Vapour pressure @ – 12 °C (μBar)	Solubility @ – 2.4 °C ($g\ L^{-1}$)		log K_{OW} (– 12 °C)
			Freshwater[4]	Seawater (S = 35)[5]	
Naphthalene	128.2	101.0[a]	1.7×10^{-1}	1.2×10^{-1}	3.3[5]
Phenanthrene	178.2	0.40[a]	1.7×10^{-2}	1.1×10^{-2}	4.4[5]
Pyrene	202.3	0.06[a]	6.4×10^{-3}	4.1×10^{-3}	4.9[6*]
Benzo(a)pyrene	252.3	1.20[a]	1.2×10^{-3}	7.0×10^{-4}	5.7[6*]

* *Values at 0 °C.*

Table S2. Filtration recoveries.

PAH	Representative surrogate	Recovery (%) (n=6)
Naphthalene (NAP)	NAP-D_8	73 ± 15
Phenanthrene (PHE)	PHE-D_{10}	87 ± 8
Pyrene (PYR)	PYR-D_{10}	84 ± 11
Benzo(a)pyrene (BaPYR)	BaPYR-D_{12}	91 ± 13

Table S3. Ions targeted during the GC-MS analysis with the SIM mode.

Compound	Qualifier	Quantifier 1	Quantifier 2
Naphthalene	128	108	102
Naphthalene-D8	136	137	134
2-Fluorobiphenyl	172	152	170
Phenanthrene	178	176	152
Phenanthrene-D10	188	184	160
Pyrene	202	200	101
Pyrene-D10	212	208	106
Benzo(a)pyrene	252	250	126
Benzo(a)pyrene-D12	264	260	

Table S4. Background concentrations of PAHs and particles in the microcosms and blanks.

Parameter	NAP (ng L^{-1})	PHE (ng L^{-1})	PYR (ng L^{-1})	BaPYR (ng L^{-1})	Background particles (mg L^{-1})
Experimental seawater blanks (n=10)	n/d	n/d	n/d	n/d	0.7 ± 0.2
Control seawater blanks (n=3)	n/d	n/d	n/d	n/d	0.6 ± 0.3
Control ice blanks (n=3)	n/d	n/d	0.8 ± 0.2	n/d	0.9 ± 0.4
Field blanks	n/d	1.1 ± 0.2	1.5 ± 1.1	1.4 ± 0.1	n/d
Laboratory blanks (n=14)	0.6 ± 0.4	0.7 ± 0.2	1.1 ± 0.2	1.3 ± 0.5	n/d

n/d: not detected

Table S5. Solid-phase extraction and ultra-sonication recoveries and method detection limits.

Parameter	NAP	PHE	PYR	BaPYR
Solid-phase extraction/liquid recovery (%)	24 ± 7%	67 ± 7%	75 ± 9%	91 ± 11%
Ultrasonication/particulate recovery (%)	77 ± 14%	87 ± 13%	95 ± 9%	101 ± 8%
Method Detection Limit (ng L^{-1})	0.9	1.1	1.2	2.1

Table S6. Mean and standard deviations (n=3) total masses of the four PAHs throughout the experiment. Day 0 corresponds to the day of PAH mixture injection.

DAY	NAP (µg)	PHE (µg)	PYR (µg)	BaPYR (µg)
0	511	505	498	506
2	460±11	501±8	495±10	502±6
5	419±22	497±25	490±26	504±11
9	360±15	468±20	489±24	501±11
14	288±14	449±18	484±24	499±13
19	213±9	417±17	474±12	501±10

Table S7. Mean apparent distribution coefficients (log K_d) and standard deviations for the four PAHs in the ice and the seawater media over time.

	Sea Ice				Seawater			
Day	NAP	PHE	PYR	BaPYR	NAP	PHE	PYR	BaPYR
2	3.8 ± 0.3	3.7 ± 0.1	5.1 ± 0.1	5.8 ± 0.2	2.3 ± 0.0	3.4 ± 0.2	4.6 ± 0.1	6.2 ± 0.1
5	3.9 ± 0.1	4.8 ± 0.3	5.4 ± 0.2	6.3 ± 0.1	2.2 ± 0.1	4.4 ±0.2	4.5 ± 0.1	5.7 ± 0.0
9	3.9 ± 0.2	4.7 ± 0.3	5.5 ± 0.1	6.3 ± 0.1	2.3 ± 0.1	4.8 ± 0.1	5.0 ± 0.0	6.0 ± 0.1
14	4.1 ± 0.1	4.9 ± 0.3	6.2 ± 0.1	6.5 ± 0.1	2.4 ± 0.1	3.5 ± 0.1	4.0 ± 0.0	4.8 ± 0.1
19	4.5 ± 0.0	5.7 ± 0.1	6.3 ± 0.0	6.7 ± 0.1	2.4 ± 0.1	3.9 ± 0.2	4.5 ± 0.0	5.2 ± 0.4

Table S8. Average ice thickness and ice growth rate throughout the experiment.

	Ice formation (Feb 24)	Day 0	Day 2	Day 5	Day 9	Day 14	Day 19
Average ice thickness (cm)	0	8.0	12.5	14.3	14.8	17.3	19.3
Average ice growth rate (cm/day)	0	4.0	2.3	0.6	0.1	0.5	0.4

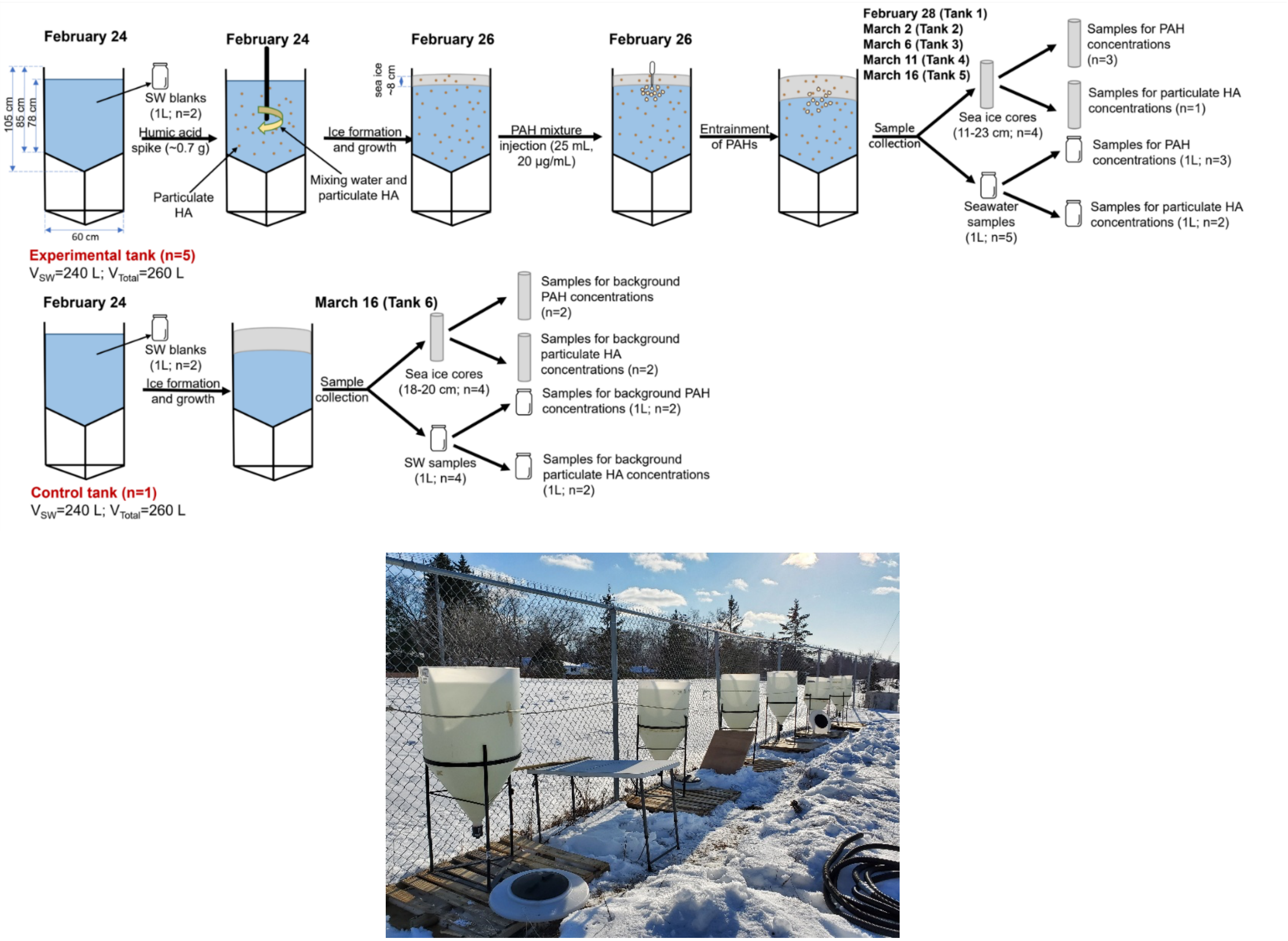

Figure S1. Schematic of the experimental design, key steps of the study, and sample collection plan (upper panel), and a picture of the microcosm set-up (lower panel).

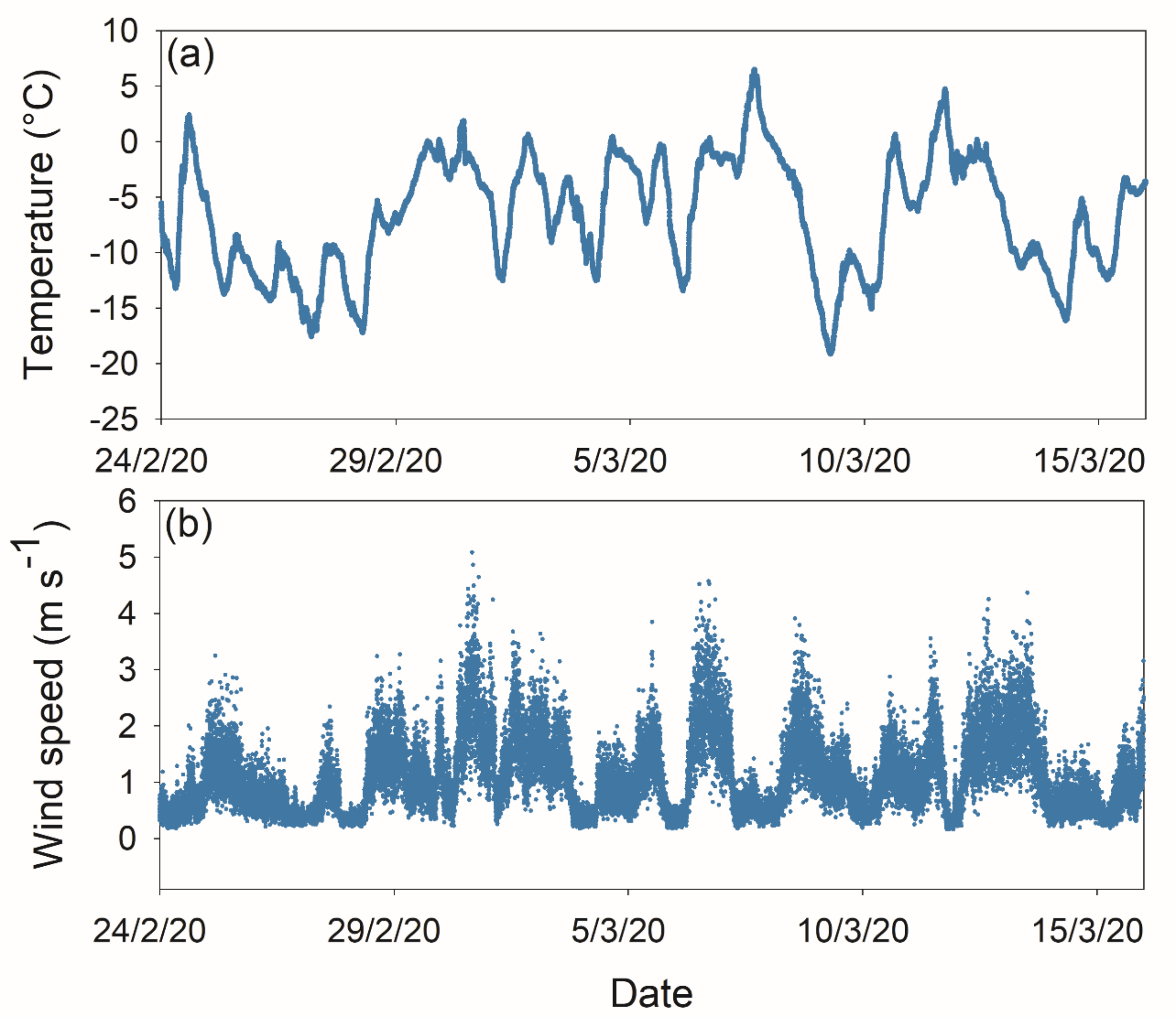

Figure S2. Variabilities of the *in situ* a) ambient air temperature and b) wind speed during the experiment, as measured at 1.5 m above the ground.

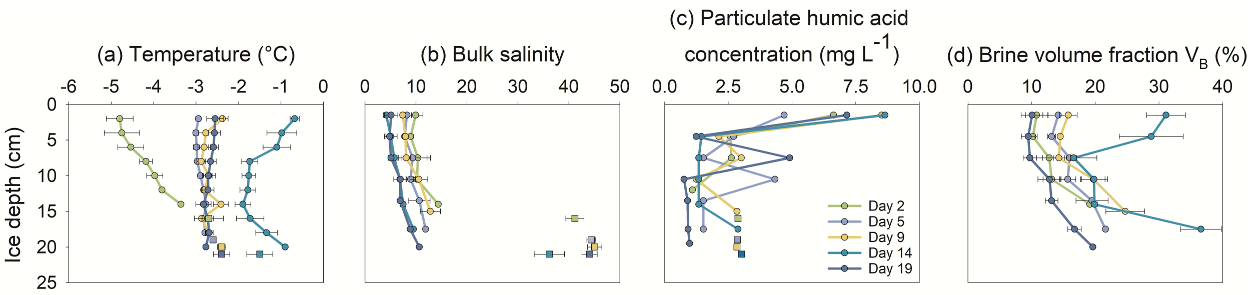

Figure S3. Vertical profiles of a) temperature, b) bulk salinity, c) concentration of particulate humc acid (HA), and d) brine volume fraction across the bulk ice (circle symbols) and in the water column (square symbols) in the experimental microcosms at various days after the addition of particulate HA and PAHs. The uppermost point on the y-axis corresponds to the atmosphere-sea-ice interface. Note that the particulate HA concentration in seawater on Day 14 overlaps with the one of Day 19, hence it is not visible.

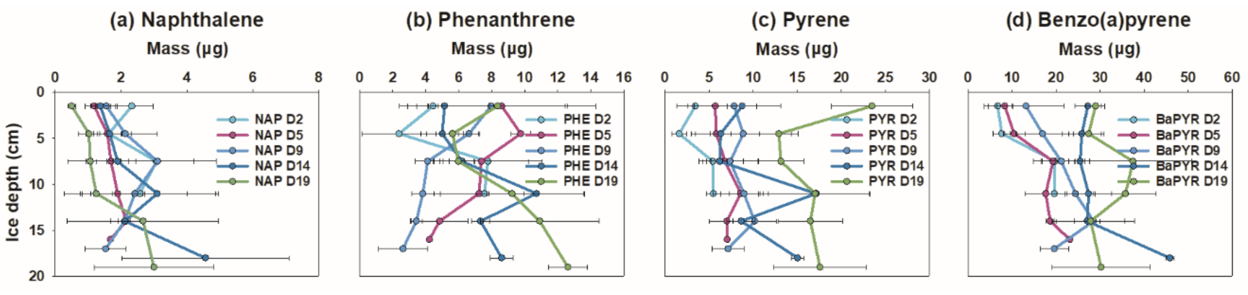

Figure S4. Vertical profiles of the respective PAHs in the ice column throughout the experiment.

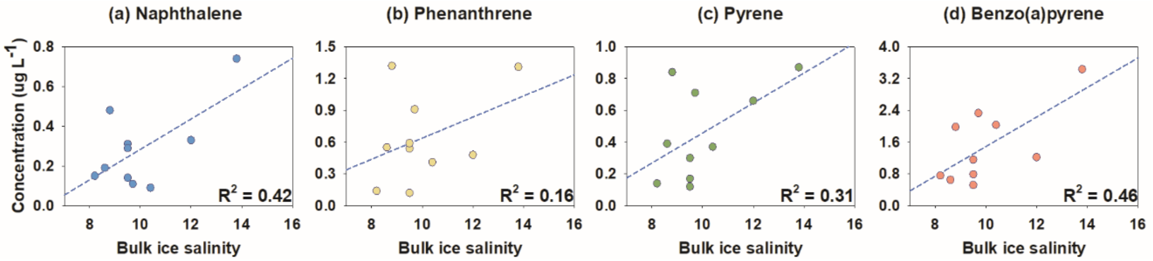

Figure S5. Relationship between the bulk salinity of sea ice and the total concentrations of the PAHs in young experimental sea ice on Day 2.

References:

1. Petrich, C.; Eicken, H., Overview of sea ice growth and properties. In *Sea Ice. 3rd Ed*, Thomas, D. N., Ed. Wiley-Blackwell: Chichester, UK, 2017; pp 1–41.
2. Notz, D.; Worster, M. G., Desalination processes of sea ice revisited. *J. Geophys. Res Oceans* **2009,** *114, C05006.*
3. Wang, F.; Pućko, M.; Stern, G., Transport and transformation of contaminants in sea ice. In *Sea Ice. 3rd ed.*, Thomas, D. N., Ed. Wiley-Blackwell: Oxford, UK, 2017; pp 472–491.
4. Saltymakova, D.; Desmond, D. S.; Isleifson, D.; Firoozy, N.; Neusitzer, T. D.; Xu, Z.; Lemes, M.; Barber, D. G.; Stern, G. A., Effect of dissolution, evaporation, and photooxidation on crude oil chemical composition, dielectric properties and its radar signature in the Arctic environment. *Mar. Pollut. Bull.* **2020,** *151*, 110629.
5. Desmond, D. S.; Saltymakova, D.; Smith, A.; Wolfe, T.; Snyder, N.; Polcwiartek, K.; Bautista, M.; Lemes, M.; Hubert, C. R. J.; Barber, D. G.; Isleifson, D.; Stern, G. A., Photooxidation and biodegradation potential of a light crude oil in first-year sea ice. *Mar. Pollut. Bull.* **2021,** *165*, 112154.
6. Meyer, T.; Lei, Y. D.; Wania, F., Measuring the release of organic contaminants from melting snow under controlled conditions. *Environ. Sci. Technol.* **2006,** *40*, 3320-3326.
